# Wide Cytokine Analysis in Cerebrospinal Fluid at Diagnosis Identified CCL-3 as a Possible Prognostic Factor for Multiple Sclerosis

**DOI:** 10.3389/fimmu.2020.00174

**Published:** 2020-03-05

**Authors:** Marco Puthenparampil, Erica Stropparo, Sofia Zywicki, Francesca Bovis, Chiara Cazzola, Lisa Federle, Francesca Grassivaro, Francesca Rinaldi, Paola Perini, Maria Pia Sormani, Paolo Gallo

**Affiliations:** ^1^Department of Neurosciences DNS, Multiple Sclerosis Centre, Università degli Studi di Padova, Padua, Italy; ^2^Biostatistics Unit, Department of Health Sciences (DISSAL), University of Genoa, Genoa, Italy; ^3^Multiple Sclerosis Centre, ULSS8 Berica, Ospedale San Bortolo, Vicenza, Italy

**Keywords:** cytokine, cerebrospinal fluid, multiple sclerosis, CCL-3, MIP-1alfa

## Abstract

**Background:** Apart from IgG oligoclonal bands, no other biomarker has, to date, been validated for diagnostic and/or prognostic purposes in multiple sclerosis (MS).

**Aim:** To investigate a wide panel of cytokines and chemokines in the cerebrospinal fluid (CSF) of relapsing–remitting MS (RRMS) patients and evaluate their association with clinical and magnetic resonance imaging (MRI) parameters, as well as their predictive clinical value.

**Methods:** Fifty-one RRMS at clinical onset and 17 other not inflammatory neurological disorders (ONINDs) underwent brain MRI (including 3D-T1, 3D-FLAIR, and 3-DIR sequences) and CSF examination. Eighty-seven cytokines and chemokines were analyzed in CSF by Multiplex technology.

**Results:** Compared to ONIND, CXCL-10, CXCL-11, CXCL-13, CCL-1, CCL-2, CCL-3, CCL-22, IL-16, and BAFF were significantly (*p* < 0.05) increased in RRMS CSF. However, only CCL-3 was associated with both MS diagnosis and IgGOB detection. Based on a 95%CI in ONIND (cut-off value: 0.798 pg/ml) and ROC analysis (cut-off value: 0.495 pg/ml), RRMS patients were stratified in CCL-3^high^ (>0.736 pg/mL), CCL-3^medium^, and CCL-3^low^ (<0.495 pg/ml). Survival analysis disclosed a strong association between high CCL-3 values and disease reactivation (OR = 4.9, 95%CI: 1.8–13.3, *p* < 0.005) in the following 2 years.

**Conclusions:** CCL-3 deserves further investigation as a candidate prognostic biomarker for RRMS.

## Introduction

Since the earliest disease phases, multiple sclerosis (MS) brain is characterized by a mix of white and gray matter inflammation and neuroaxonal damage and loss (1, 2). Attempts aimed at discovering intrathecally produced biomarkers of inflammation or neurodegeneration gave inconsistent results and showed several methodological limitations (3, 4). Indeed, except for IgG oligoclonal bands (5), up to date, no biomarker, among those suggested to hold the potential for predicting disease course in MS, has been validated for clinical purposes.

Cytokines and chemokines are the most investigated biomarkers of brain inflammation. These soluble factors, which mainly act in paracrine or autocrine manners, constitute a complex, continuously changing and unpredictable network, mainly characterized by redundancy and pleiotropism (6). In the central nervous system (CNS) of MS patients, the cellular sources of cytokines/chemokines may be immune cells, astrocytes, and microglia. Thus, the identification of the specific cellular source of a given cytokine/chemokine and its activity within the MS neuro-immunological network is quite a hard task. In addition, immunomodulatory/immunosuppressive therapies have an impact on cytokines/chemokines production, even within the CNS. Hence, if a possibility exists to detect a potential biomarker of inflammation that can be used for clinical purposes, the ideal condition would be to analyze the largest panel of cytokines/chemokines in the cerebrospinal fluid (CSF) of newly diagnosed and untreated patients.

In this study, we investigated the presence and concentration of 87 cytokines in MS CSF obtained at clinical onset and evaluated their correlation with magnetic resonance imaging (MRI) parameters of white matter (WM) and gray matter (GM) damage. Moreover, the majority of our MS patients completed a 24-months clinical and radiological follow-up, giving the chance of evaluating the prognostic value of these molecules.

## Materials and Methods

### Study Population

To ensure the inclusion of MS patients with no evidence of CSF-restricted IgG oligoclonal bands, 51 not-consecutive relapse-onset MS (RRMS) patients at the time of the diagnosis, and 17 other not inflammatory neurological disorder (ONIND) were included in this study. MS diagnosis was achieved in agreement with the most recent diagnostic criteria (7). Brain (see below) and spinal cord MRI, CSF examination (see below), visual evoked potential, serum biochemical analysis, and immunological screening were performed in all subjects. Exclusion criteria were the presence of comorbidities and previous treatment with drugs affecting the immune system. Expanded disability status scale (EDSS) was assessed by trained physicians (RF, FL, PP). The ONIND group was constituted by subjects complaining tension headache, transient subjective sensory symptoms and psychosomatic disorders, as well as unspecific white matter alterations who underwent a detailed diagnostic workup including routine blood tests, B12 vitamin, folates, and angiotensin converting enzyme (ACE) concentrations, as well as immunological screening (detecting ANA, ANCA, ENA, anti-dsDNA, anti-β_2−_glycoproteinI, anti-cardiolipin, and LAC), CSF examination, and brain and spinal cord MRI to exclude neurological disorders. Even if no evidence of neurological or systemic diseases was achieved in these subjects, these patients were defined as ONIND.

The study was approved by the Ethics Committee of the Azienda Ospedaliera di Padova. Written informed consent was obtained from patients and controls.

### Routine CSF Analysis

Paired CSF and serum specimens were collected by non-traumatic lumbar puncture between 8.00 and 9.00 a.m. Routine examination included (1) cell count and differentiation, (2) the calculation of the CSF/serum albumin ratio (Q_Alb_, to estimate the integrity of the blood–brain barrier, BBB), (3) the calculation of the IgG index (8) and the IgG hyperbolic function to obtain the IgG intrathecal synthesis fraction (IgG_IF_) and the local production (IgG_LOC_) (9), and (4) the demonstration of IgG oligoclonal bands (IgGOB) by means of agarose isoelectric focusing followed by transfer to nitrocellulose membrane, IgG specific immunofixation, amplification with avidin-biotin, and peroxidase staining (10). BBB damage was defined as a Q_Alb_ above the normal value corrected for the patient's age (i.e., age/15+4, Qlim_Alb_). CSF aliquots were stored at −80°C until further analysis.

### Cytokine Investigation

CSF concentration of 87 cytokines was assessed by Multiplex technology (Bio-Plex Pro Human Cytokine, GF and Diabetes 27-Plex Panel, Bio-Plex Pro Human Chemokines 40-Plex Panel, Bio-Plex Pro Human Inflammation Assays 37-Plex Panel). For each molecule, the percentage of detectable concentration was evaluated. Cytokines detected in <50% of all (MS and ONIND) samples were excluded from the analysis (Supplementary Material 1). When the same cytokine was detectable by two kits, results from the kit with higher sensitivity and frequency of detection were considered.

### MRI Protocol

Images were acquired using a 3-T scanner (Ingenia, Philips Medical Systems, Best, The Netherlands) with 33-mT/m power gradient and a 32-channel head coil. No major hardware upgrades occurred during the study, and bimonthly quality-assurance sessions assured measurement stability. The following sequences were acquired: (a) three-dimensional (3D) turbo field echo (TFE, 3D-T1): repetition time (RT) 7.8 ms; echo time (ET) 3.6 ms; 180 contiguous axial slices with the off-center positioned on zero with thickness of 1.0 mm; flip angle = 8°; matrix size = 220 × 220; FOV = 220 × 220 × 180 mm^3^. This sequence was acquired before and after gadolinium administration. (b) 3D-fluid attenuated inversion recovery (FLAIR): RT 4,800 ms; ET 310 ms; inversion time (IT) 1,650 ms; 365 contiguous axial slices with thickness of 1.0 mm; matrix size 256 × 256; and FOV = 256 × 256 × 182 mm^3^. (c) 3D-double inversion recovery (DIR): RT 13,000 ms, ET 10 ms, IT 3,400/325 ms; 40 contiguous axial slices, resolution 1 × 1 × 3 mm; FOV 230 × 200 mm; time 3.5 min. Two experienced observers (SZ, AL), blinded to the patient's identity, assessed all images. Global cortical thickness (gCTh) was analyzed by means of Freesurfer on 3D-T1 sequences. WM lesions were identified on FLAIR sequences, while cortical lesions (CLs) were identified on DIR scans by two blinded evaluators (ZS, CC) using published consensus recommendations (11).

### Follow-Up Protocol

During the 24-months follow-up, clinical evaluations with EDSS assessment were performed every 6 months, while the brain MRI was performed annually. In the event of relapse, defined as the occurrence of new symptoms or exacerbation of existing symptoms that lasted for 24 h or longer, in the absence of concurrent illness or fever, and occurring 30 days or more after a previous relapse, a further clinical evaluation was performed.

During the follow-up, a disease reactivation was defined radiologically, in the presence of new/enlarging white matter lesions, and clinically, in the case of disease relapse or a sustained progression of disability based on 1-step EDSS progression (for EDSS ≤ 5.5) or 0.5-step EDSS progression (for EDSS > 5.5) confirmed at two consecutive examinations at least 12 months (12).

### Statistical Analysis

Quantitative data are presented as medians (1st and 3rd quartile) or means (±standard deviation) and categorical data as absolute numbers and percentages. Differences between groups (ONIND and MS) were assessed by Mann–Whitney *U* or *t*-test according to the distribution of the variables. For ordinal variables, Pearson's chi-square test was used. To avoid collinearity, the correlation coefficients between all the cytokines examined were calculated. Multivariate logistic regression was used to find relevant independent explanatory cytokines for MS patients. Only factors significantly associated with the outcome at univariate analysis were included in a multivariate model with a stepwise procedure. For CCL-3 cut-off, two approaches were applied: a formula considering ONIND values (μ ± 1.96^*^σ) exclusively and a receiver operating characteristic curve (ROC curve), which considered all (ONIND and MS) values, balancing between true positive result (MS) and false positive result (not-MS) at every possible decision boundary. *P* < 0.05 were considered statistically significant. All analyses were carried out using the SAS software version 9.3 (Institute Inc., Cary, NC, USA).

## Results

### Study Populations: Demographic, Clinical, and CSF Findings

RRMS patients and ONIND did not differ in any demographic parameter. CSF-restricted IgGOB (IgGOB+) and increased IgG indexes were demonstrated in 41/51 (80.4%) and 19/51 (37.3%) RRMS patients, respectively. Eleven RRMS had normal IgG IEF pattern (IgGOB-) and normal IgG indexes. A very mild BBB damage was observed in 8/51 patients (15.7%). No evidence of intrathecal IgG synthesis was observed in ONIND, but one patient had a mild BBB damage (Q_Alb_/Q_albLIM =_ 1.3) (Table 1).

**Table 1:** Demographic, standard cerebrospinal fluid, and MRI parameters.

	**Healthy controls *N* = 17**	**Relapsing–remitting MS *N* = 52**	***p*-value**
Gender (Female)	12 (70.6%)	38 (73.1%)	1.0
Age (y)	44.8 ± 8.1	35.7 ± 9.1	0.06
Disease duration (m)	n.a.	13.4 ± 23.1	n.a.
Q_alb_	4.8 ± 1.8	5.0 ± 1.8	0.8
BBB damage	1 (5.9%)	8 (15.4%)	0.4
IgG_S_ (g/L)	11.0 ± 2.2	10.3 ± 2.0	0.2
IgG_L_ (g/L)	0.02 ± 0.01	0.03 ± 0.01	0.0070
IgG Index	0.48 ± 0.05	0.71 ± 0.29	0.0017
IgG_LOC_ (mg/dl)	0.0 ± 0.0	0.47 ± 0.95	0.046
IgG_IF_	0.0 ± 0.0%	10.6 ± 18.1%	0.019
IgGOB	0 (0%)	41 (78.8%)	n.a.
WMLV (mm^3^)	n.a.	868.6 ± 1073.4	n.a.
WMLn	n.a.	45.2 ± 38.4	n.a.
GMLV	n.a.	53.5 ± 50.8	n.a.
GMLn	n.a.	7.4 ± 14.4	n.a.
CTh mm	2.44 ± 0.07	2.48 ± 0.09	0.206

*Data are expressed as mean (±standard deviation) or frequencies (percentage). y, years; m, months; MS, multiple sclerosis; BBB, blood–brain barrier; IgGOB, IgG oligoclonal bands; WMLV, white matter lesion volume; WMLn, white matter lesion number; GMLV, gray matter lesion volume; GMLn, gray matter lesion number; CTh, cortical thickness*.

### CCL-2 and CCL-3 CSF Concentrations Discriminate MS From ONIND

**Figure 1:**
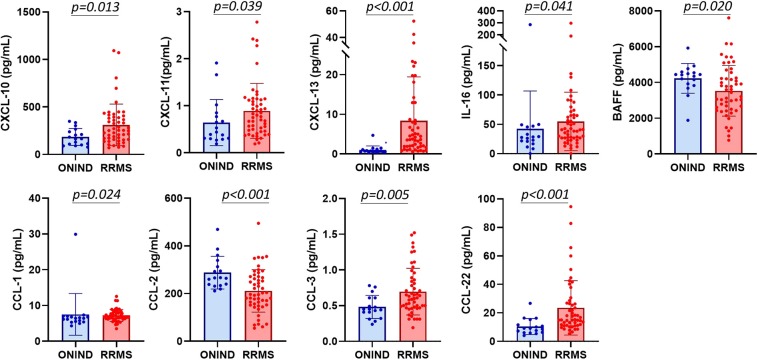
CXCL-10, CXCL-11, CXCL-13, IL-16, BAFF, CCL-1, CCL-2, CCL-3, and CCL-22 CSF concentrations differ between other not-inflammatory neurological disorders and relapsing–remitting MS patients.

Out of 87 investigated cytokines and chemokines, CXCL-10, CXCL-11, CXCL-13, CCL-1, CCL-2, CCL-3, CCL-22, IL-16, and BAFF CSF concentrations significantly differed between RRMS patients and ONIND (Figure 1 and Supplementary Material 2). Following the univariate analysis (Supplementary Material 3), the multivariate analysis disclosed that CCL-2 and CCL-3 are significantly associated to the diagnosis of MS [OR: 0.98, 95% confidence interval (_95%_CI): 0.96–0.99, *p* = 0.001, and OR > 1,000.0, _95%_CI: 67.45–1,000.0, *p* = 0.0008, respectively].

### BAFF Associates With Intrathecal IgG Synthesis

In MS patients, only CSF concentration of BAFF and CCL-3 associated with both IgGIF (OR: 1.0, *p* = 0.0037 and OR: 1,000.0, *p* = 0.008) and IgGOB (OR: 1.0, *p* = 0.039 and OR: 1,000.0, *p* = 0.021) (Supplementary Material 4). Finally, when evaluating only MS patients with positive IgGIF, this association was confirmed for BAFF (IgG index: r: −0.69, *p* < 0.001) but not for CCL-3 (r: −0.39, *p* = 0.086).

### Only CCL-3 and CXCL6 Associate With MRI Parameters at Baseline

We investigated the possible correlations between the eight cytokines detected in RRMS CSF with the MRI parameters at baseline. Only CXCL-6 levels inversely, but very mildly, associated with CTh (r: −0.30, *p* = 0.048). No further correlation was observed between CSF and MRI parameters. A mild association was found between CCL-3 levels and the WM lesion volume, since MS patients with the highest WMLV had also higher CCL-3 CSF concentrations (0.79 ± 0.35 vs. 0.59 ± 0.79, *p* = 0.03).

### CCL-3 as Candidate Prognostic Biomarker

The association of CCL-3 with the diagnosis of MS prompted us to investigate the prognostic value of this molecule. Thirty-nine MS patients (76.5%) were clinically and radiologically monitored for a mean period of 36.4 ± 7.7 months (range 26–51 months). Thirty-four initiated therapy with disease-modifying drugs within 6 months after diagnosis (Table 2). During follow-up, 13/34 (32.8%) had clinical relapses associated with MRI evidence of disease activity, while only 2/34 (5.8%) had only MRI evidence of disease activity.

**Table 2:** Demographic, clinical, and standard cerebrospinal fluid in followed-up relapsing–remitting MS (RRMS).

	***RRMS-1α^*low*^ N* = 9**	***RRMS-1α^*medium*^ N* = 15**	***RRMS-1α^*high*^ N* = 15**	***p-value***
Gender (Female)	7 (77.8%)	10 (66.7%)	11 (73.3%)	0.83
Age (y)	31.11 ± 9.66	34.93 ± 8.29	37.07 ± 8.47	0.28
Disease duration (m)	10.00 ± 22.56	9.40 ± 10.78	12.20 ± 17.65	0.89
ARR	1.00 ± 0.50	0.93 ± 0.46	1.40 ± 0.63	0.06
EDSS	1.5 (1.0–4.0)	1.5 (1.0–4.0)	2.0 (1.0–5.0)	0.92
IgGOB	6 (66.7%)	11 (73.3%)	14 (93.3%)	0.22
IgGIndex	0.59 ± 0.14	0.76 ± 0.35	0.73 ± 0.21	0.27
BBB	2 (22.2%)	3 (20.0%)	3 (20.0%)	0.99
Treatments				0.73#
None	1 (11.1%)	2 (13.3%)	0 (0%)	−
Total first line	7 (77.8%)	10 (66.7%)	14 (93.3%)	0.34#
*Glatiramer acetate*	*0 (0%)*	*5 (33.3%)*	*2 (13.3%)*	-
*Teriflunomide*	*0 (0%)*	*1 (6.7%)*	*3 (20.0%)*	-
*Dimethyl-Fumarate*	*3 (33.3%)*	*2 (13.3%)*	*6 (40.0%)*	-
*Interferon*	*4 (44.4%)*	*2 (13.3%)*	*3 (20.0%)*	-
Total Second Line	1 (11.1%)	3 (20.0%)	1 (6.7%)	0.91#
*Natalizumab*	1 (11.1%)	2 (13.3%)	0 (0%)	
*Alemtuzumab*	0 (0%)	1 (6.7%)	1 (6.7%)	-

*Considering the discrepancy between these cut-offs, MS patients were divided in RRMS-1αlow (<0.495 pg/ml, nine patients), RRMS-1αmedium (0.495–0.736 pg/ml, 15 patients), and RRMS-1αhigh (>0.736 pg/ml, 15 patients). ARR, annualized relapse rate; EDSS, Expanded Disability Status Scale. Other abbreviations as in Table 1. #: to perform chi square, all null values were set at 1. The drug are reported in italics, while the type (first or second line) not*.

**Figure 2:**
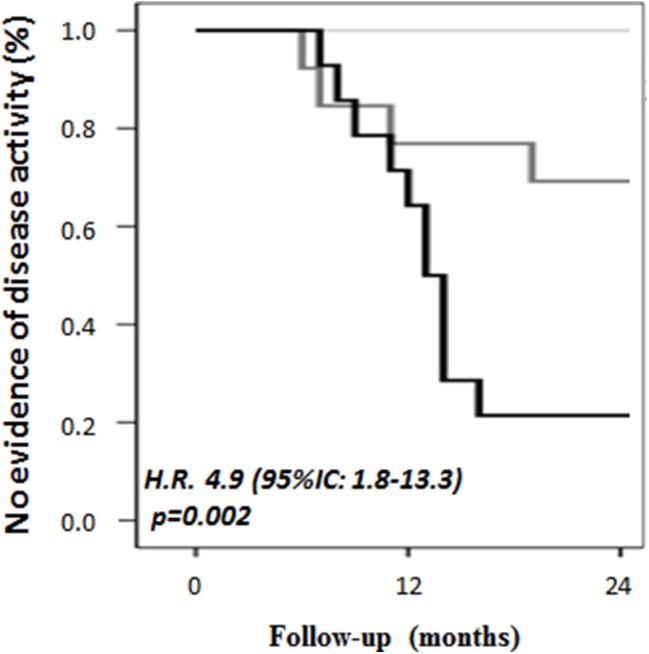
CSF CCL-3 associates with a higher probability of disease activity during follow-up. RRMS patients were divided into three groups based on ROC cut-off and on the cut-off determined by the 95%CI upper limit calculated on ONIND (i.e., μ ± 1.96*σ, i.e., 0.798 pg/ml). RRMS_CCL−3_^low^ (<0.495 pg/ml) did not present any relapse in the following 24 months, while RRMS_CCL−3_^medium^ (0.495–0.798 pg/ml) and RRMS_CCL−3_^high^ (>0.798 pg/ml) had a significantly higher rate of disease activity (*p* = 0.002).

ROC analysis identified 0.495 pg/ml as the cut-off value (AUC, 0.728; sensitivity: 71.2%, specificity 70.6%) to discriminate between ONIND and MS. Besides, the sensitivity and specificity of the 95%CI upper limit calculated on ONIND (i.e., μ±1.96^*^σ, i.e., 0.798 pg/ml) were 34.0 and 91.0%, respectively. Considering the discrepancy between these cut-offs, MS patients were divided in RRMS_CCL−3_^low^ (<0.495 pg/ml, 9 patients), RRMS_CCL−3_^medium^ (between 0.495 and 0.798 pg/ml, 15 patients), and RRMS_CCL−3_^high^ (>0.798 pg/ml, 16 patients). No difference was observed for any demographic, clinical, and radiological parameters between groups at baseline (Table 2). Survival analysis significantly differed within MS subgroups (*p* < 0.001) (Figure 2). Indeed, Cox regression analysis revealed an association between group and disease reactivation (OR = 4.9, 95%CI: 1.8–13.3, *p* = 0.002). No other baseline parameter (EDSS, disease duration, age and annualized relapse rate, IgGOB detection) or treatment was found to associate with disease reactivation.

## Discussion

The availability of many effective treatments for MS requires strategies to identify, at disease onset, patients with high probability of disease activity/worsening, in order to prescribe the most effective treatments as soon as possible. In line with this clinical need, diagnostic and prognostic relevance of many CSF molecules, especially cytokines, have been tested in MS (13, 14). However, taking into account that cytokines are characterized by five attributes (namely, pleiotropy, redundancy, synergy, antagonism, and cascade induction), the unique strategy to identify those that associate at best with MS consists in the simultaneous evaluation of the widest range of these molecules. Although the absence of a cohort of other inflammatory neurological disorders does not support us in evaluating the role of these molecules in MS diagnosis, the clinical and radiological follow-up of the majority of our patients allows us to evaluate their putative role as prognostic biomarkers.

From the 87 cytokines studied, only nine cytokines/chemokines were expressed at different levels in the CSF of RRMS at clinical onset, namely, CXCL-10, CXCL-11, CXCL-13, CCL-1, CCL-2, CCL-3, CCL-22, IL-16, and BAFF. Interestingly, all these molecules are involved in leukocyte (T- and B-lymphocytes, neutrophils, and monocyte) recruitment/trafficking, strongly supporting that leukocyte migration into the MS brain is driven by the local production of a pattern of specific recruiting molecules since the very early disease phases.

CXCL-10, CXCL-11, CCL-1, CCL-22, and IL-16 play a major role in T lymphocyte trafficking and activation. CXCL-10 and CXC-L11 (15) have also been associated to the recruitment of plasmablasts into inflammatory sites, and these cells were found increased in MS CSF (16). In addition, CXCL-10 and CXCL-11, induced by inflammatory stimuli, have been suggested to play a role in the maintenance of intrathecal inflammation (15). CCL-1, mainly produced by activated T lymphocytes, up-regulates Treg functions and enhances microglia proliferation and phagocytosis (17, 18). IL16, a cytokine produced by antigen-presenting cells (APCs, especially dendritic cells, macrophages, microglia) (19), attracts T-helper lymphocytes (20) and modulates APC-T cell interaction (20, 21). *In vitro*, IL-16 activates microglial cells, inducing the release of cytokines (i.e., IL-16, IL-1β, TNF-α, IL-6) that are involved in the stimulation and maintenance of M1 microglial phenotype (22).

As expected, we further confirmed the increased intrathecal synthesis of CXCL-13, thus pointing out a role of this chemokine on MS immunopathology. Interestingly, CXCL-13 might play a relevant role in MS pathogenesis, since it associates with intrathecal IgGOB synthesis (23) and lymphocyte recruitment (both B- and T-cells) (24, 25). Moreover, its increased concentration in the CSF reflects pathological MS findings (16, 26), especially the GM demyelination and cortical inflammation. On the base of the highly convergent and convincing literature data, it is probably time to consider this chemokine as a possible marker of intrathecal inflammation, and a multicenter study aimed at defining its role as a prognostic marker should be seriously taken into consideration.

In addition, we confirmed in an additional (27), independent cohort that BAFF is decreased in the CSF of early MS. As already reported, this cytokine could enhance the survival of infiltrating plasmacells/plasmablasts (14).

No data are currently available on the intrathecal source of CXCL-6, a chemokine primarily involved in neutrophil recruitment. Although neutrophils seem not to be involved in MS pathology, a hypothetical role of neutrophils in MS has been suggested. Indeed, many molecules (G-CSF, CXCL-1, CXCL-8, CXCL-5, neutrophil-elastase), potentially acting on neutrophils, are expressed in the MS brain, CSF (28), and blood. Moreover, blood-derived neutrophils of MS patients are ready to fully respond after stimulation, and the administration of G-CSF exacerbates RRMS (29). Finally, gain-of-function mutations in the gene encoding pyrin, a protein associated with innate immune response, were associated with a worse MS course (30). It has to be pointed out that the mild inverse correlation observed between CXCL6 and CTh has to be considered with extreme caution and needs to be further confirmed in a larger number of patients.

The most interesting finding of our study was the significantly increased expression CCL-2 and especially CCL-3 in RRMS CSF. These cytokines were associated with MS diagnosis and WM lesion volume and were predictive of disease activity. The increased CCL-3 concentration in MS CSF, already noticed several years ago (31), is in line with several experimental and histological evidences. First, *in vitro* experiments showed that the pro-inflammatory cytokines IL-1β and TNF-α, expressed in MS lesions, induce the transcription of CCL-3 mRNAs in human astrocytes. Second, CCL-3 is a chemotactic factor for monocyte-derived dendritic cells (32) and CD3^+^CCR5^+^ T lymphocytes (33), cells that may play a role in MS pathology. Finally, microglial cells in the normal-appearing white matter of MS patients expressed CCL-3, whose concentration increases during an inflammatory response (33, 34). Thus, considering that CCL-3 marks glial cell activation, an increased CSF level of this cytokine in RRMS is not surprising since astrocyte and microglia activation and proliferation constitute a major histological feature of white and gray matter inflammation in MS (35). Moreover, these observations are in line with the strong association between the CSF levels of CCL-3 and the risk of disease reactivation in the next 2 years observed in our MS cohort. However, it is not clear whether CCL-3 reflects ongoing mechanisms of damage or might play a detrimental role in MS by recruiting macrophages inside the CNS. The role of macrophages in MS is strongly supported by their presence in white matter lesions (36, 37) as well as by the identification of myelin debris in their lysosomes (38). In addition, in MS pathogenesis, macrophages could act directly, engulfing oligodendrocytes by antibody/complement-dependent opsonization of target cells, and pattern recognition receptors (such as scavenger receptors) (39), or indirectly, producing pro-inflammatory cytokines (IL-1β, IL-6, IL-8, IL-12, TNF-α) (40).

We are aware that our study has some limitations. First is the relatively limited number of patients included in the study due to the very selective criteria of inclusion, aimed at obtaining a highly homogeneous cohort of patients. Second is the lack of serum data that did not allow the calculation of cytokine indexes. However, it has to be pointed out that the CSF concentrations of intrathecally synthesized cytokines/chemokines, their autocrine/paracrine mechanism of action, and the CSF/serum protein gradient make unlikely their detection in serum; thus, we believe that our study lacks sensitivity rather than specificity. Third is the absence of longitudinal CSF data, which, associated with clinical data, may better give the opportunity of exploring the prognostic relevance of a putative biomarker. However, serial lumbar punctures are usually not performed for clinical purposes and not admitted for research purposes by our Ethic Committee.

Taken all together, the nine cytokines/chemokines that we found in RRMS CSF at clinical onset form a quite homogeneous network of soluble factors that promote and modulate the migration of leukocytes into the CNS and the proliferation of glial cells, and, considering the features of MS histology, their detection sounds convincing. The role of CCL-3 as a potential biomarker of white matter inflammation and disease course in early RRMS phases (i.e., when the inflammatory component of MS is particularly relevant) stimulates further investigation, especially in comparison/association with other putative prognostic biomarkers, such as Neurofilaments Light (NFL) (41) and Chitinase 3-like 1 (42, 43).

## Conclusions

Our study allowed the identification of a group of cytokines/chemokines that constitute a network of homing and activating factors, which can be reasonably involved in the inflammatory process that takes place in the MS brain in early disease phases. In particular, considering the possible diagnostic and prognostic role of CCL-3, as well as its association with WM pathology, this molecule is worthy of further investigation in larger cohorts of patients.

## Data Availability Statement

The datasets generated for this study are available on request to the corresponding author.

## Ethics Statement

The studies involving human participants were reviewed and approved by the Ethics Committee of the Azienda Ospedaliera di Padova. The patients/participants provided their written informed consent to participate in this study.

## Author Contributions

MP: study concept and design, collection of clinical and immunological data, writing and critical revision of the manuscript, and statistical analysis of the data. ES and CC: collection of radiological and immunological data, writing, and critical revision of the manuscript. FB: statistical analysis of the data, writing, and critical revision of the manuscript. SZ, LF, and FR: collection of radiological data, writing, and critical revision of the manuscript. FG: writing and critical revision of the manuscript. PP: study concept and design, collection of radiological data, writing, and critical revision of the manuscript. MS and PG: study concept and design, writing, and critical revision of the manuscript.

### Conflict of Interest

MP reports grants and personal fees from Novartis, Almirall, Biogen Idec, and Sanofi Genzyme, and grants from Teva, outside the submitted work. He served as an advisory board member of Novartis, Biogen and Sanofi Genzyme. SZ reports grants from Sanofi Genzyme and Almirall, outside the submitted work. LF reports receiving personal fees from Almirall, grants from Merck Serono, grants and personal fees from Novartis, Biogen Idec, Sanofi Genzyme, and Teva, outside the submitted work. FR serves as an advisory board member of Biogen-Idec and Sanofi Genzyme, and has received funding for travel and speaker honoraria from Merck Serono, Biogen Idec, Sanofi-Aventis, Teva, and Bayer Schering Pharma. PP has received funding for travel and speaker honoraria from Merck Serono, Biogen Idec, Sanofi-Aventis, and Bayer Schering Pharma and has been consultant for Merck Serono, Biogen Idec and Teva. MS received compensation for serving on the Scientific Advisory Boards of Teva, Genzyme, Novartis, Roche and Vertex; funding for travel or speaker honoraria from Merck Serono, Teva, Genzyme, Novartis, Biogen and Roche; consultancy fees from Merck Serono, Biogen, Teva, Genzyme, Roche, GeNeuro, MedDay and Novartis; Speakers' Bureaus from Teva, Merck Serono, Biogen, Novartis and Genzyme. MPS declares personal fees from Actelion, Biogen, Genzyme, Merck, Novartis, Roche, Serono, Synthon and Teva, all unrelated to this study. PG reports grants and personal fees from Novartis, Almirall, Biogen Idec, Sanofi Genzyme, Teva, and Merck Serono, grants from the University of Padova, the Italian Ministry of Public Health, the Veneto Region of Italy, and the Italian Association for Multiple Sclerosis, outside the submitted work. The remaining authors declare that the research was conducted in the absence of any commercial or financial relationships that could be construed as a potential conflict of interest.
