# Supplementary material for: Wide Cytokine Analysis in Cerebrospinal Fluid at Diagnosis Identified CCL-3 as a Possible Prognostic Factor for Multiple Sclerosis
Source: Front Immunol. 2020 Mar 5;11:174. doi: 10.3389/fimmu.2020.00174 (PMC7066207; doi:10.3389/fimmu.2020.00174)
Supplement: Supplementary file 1 [file Data_Sheet_1.pdf]

Supplementary Materials 1. Performance of the Multiplex kits. CSF cytokines were evaluated by Bio-Plex Pro Human Cytokine, GF and Diabetes 27-Plex Panel (left columns), Bio-Plex Pro Human Inflammation Assays 37-Plex Panel (central columns), and Bio-Plex Pro Human Chemokines 40-Plex Panel (right columns). Cytokines detected in less than 50% of all (MS and ONIND) samples were excluded from the analysis (grey). Coefficient of variability (CV) is also reported in percentage

Bio-Plex Pro Human Cytokine, GF and Diabetes 27-Plex Panel

Cytokine	Detectable values (%)	CV (%)
IL-1β	30%	
IL-1Ra	100%	7,3
IL-2	4%	
IL4	61%	6,6
IL5	33%	
IL-6	29%	
IL-7	43%	
IL-8	100%	5,3
IL-9	100%	4,9
IL-10	38%	
IL-12p70	7%	
IL-13	100%	5,8
IL-15	93%	6,6
IL-17A	17%	
Eotaxin	100%	6,1
Basic FGF	46%	
G-CSF	91%	5,7
GM-CSF	75%	6,8
IFN-γ	94%	7,2
IP-10	100%	4,9
MCP-1	100%	5,7
MIP-1α	100%	6,6
MIP-1β	100%	4,9
PDGF	54%	7,1
RANTES	100%	5,7
TNF-α	67%	6,5
VEGF	100%	8,5

Bio-Plex Pro Human Inflammation Assays 37-Plex Panel

Cytokine	Detectable values (%)	CV (%)
APRIL	86%	6,6
BAFF	100%	2,8
sCD30	100%	2,3
sCD163	100%	3,9
Chitinasi3-like	35%	
sIL-6Rb	100%	4,1
IFN-α2	7%	
IFN-β	100%	4,5
IFN-γ	25%	
IL-2	3%	
sIL-6Ra	100%	3,1
IL-8	100%	5,9
IL-10	100%	12,2
IL-11	91%	5,3
IL-12p40	16%	
IL-12p70	12%	
IL-19	91%	6,2
IL-20	100%	4,3
IL-22	87%	6,1
IL-26	100%	4,8
IL-27	86%	4,7
IL-28A	4%	
IL-29	7%	
IL-32	100%	6,2
IL-34	100%	5,7
IL-35	61%	7,2
LIGHT TNFSF14	100%	5,2
MMP-1	29%	
MMP-2	26%	
MMP-3	4%	
Osteocalcin	99%	7,8
Osteopontin	97%	4,4
Pentraxin-3	100%	5,2

Bio-Plex Pro Human Chemokines 40-Plex Panel

Cytokine	Detectable values (%)	CV (%)
CCL-21	100%	11,6
CXCL-13	100%	3,6
CCL-27	100%	4,1
CXCL-25	97%	4,0
CCL-11	100%	4,5
CCL-24	100%	2,6
CCL-26	100%	3,8
CX3CL-1	100%	3,5
CXCL-6	91%	4,8
GM-CSF	100%	3,1
CXCL-1	100%	4,3
CXCL-2	88%	4,6
CCL-1	100%	4,8
IFN-γ	84%	5,5
IL-1β	14%	
IL-2	65%	4,2
IL-4	100%	7,6
IL-6	100%	3,8
IL-8	100%	3,8
IL-10	97%	5,1
IL-16	96%	4,4
CXCL-10	100%	4,0
CXCL-11	99%	4,9
CCL-2	100%	2,5
CCL-8	100%	4,0
CCL-7	100%	5,2
CCL-13	100%	5,7
CCL-22	100%	5,9
MIF	100%	4,0
CXCL-9	84%	3,8
CCL-3	100%	5,2
CCL-15	100%	4,1
CCL-20	17%	

	sTNF-R1	100%	2,3
	sTNF-R2	100%	3,4
	TSLP	100%	6,7
	TNFSF12	100%	3,6

	CCL-19	100%	4,1
	CCL-23	99%	5,3
	CXCL-16	100%	2,1
	CXCL-12	100%	4,1
	CCL-17	39%	
	CCL-25	100%	4,0
	TNF-α	100%	7,5
