# Supplementary material for: Wide Cytokine Analysis in Cerebrospinal Fluid at Diagnosis Identified CCL-3 as a Possible Prognostic Factor for Multiple Sclerosis
Source: Front Immunol. 2020 Mar 5;11:174. doi: 10.3389/fimmu.2020.00174 (PMC7066207; doi:10.3389/fimmu.2020.00174)
Supplement: Supplementary file 2 [file Data_Sheet_2.DOCX]

**Supplementary Materials 2.** **CSF cytokines in ONIND and RMS patients.** Eighty-seven cytokines were evaluated in HC and RMS patients. T-test was applied to compare normally-distributed variables. Otherwise. U-Mann Whitney test was performed. Not normally-distributed cytokines were IL-5, CCL-3, Chitinasi3-like1, IL-12p40, IL-20, IL-27, LIGHT-TNFSF14, MMP-2, sTNF-R2, CXCL13, CLL26, IL-8, IL-16, CXCL10, CXCL11, and MCP-2.

|  | **ONIND** | | | |  | | **RRMS** | | | |
| --- | --- | --- | --- | --- | --- | --- | --- | --- | --- | --- |
|  | **Average ± St. Dev.** | | | **Median** | **p-value** | **FDR** | **Average ± St. Dev.** | | | **Median** |
| **CXCL-13** | 1.02 | ± | 1.00 | 0.70 | <.0001 | <.0001 | 7.95 | ± | 10.94 | 3.71 |
| **CCL-22** | 10.40 | ± | 5.61 | 9.18 | 0.0004 | 0.014 | 23.28 | ± | 18.78 | 16.01 |
| **CCL-2** | 287.54 | ± | 68.50 | 265.95 | 0.003 | 0.072 | 210.73 | ± | 91.31 | 196.48 |
| **CCL-3** | 0.48 | ± | 0.16 | 0.45 | 0.005 | 0.090 | 0.69 | ± | 0.30 | 0.62 |
| **CXCL-10** | 184.47 | ± | 88.44 | 165.70 | 0.013 | 0.187 | 300.00 | ± | 190.23 | 254.85 |
| **BAFF** | 4,225.87 | ± | 831.73 | 4,422.15 | 0.020 | 0.240 | 3,486.86 | ± | 1,317.93 | 3521.43 |
| **CCL-1** | 7.52 | ± | 5.82 | 6.40 | 0.024 | 0.247 | 7.22 | ± | 1.64 | 6.80 |
| **CXCL-11** | 0.64 | ± | 0.49 | 0.44 | 0.039 | 0.328 | 0.87 | ± | 0.55 | 0.79 |
| **IL-16** | 42.53 | ± | 64.17 | 27.63 | 0.041 | 0.328 | 55.31 | ± | 51.16 | 41.07 |
| **CCL-27** | 6.54 | ± | 4.89 | 5.84 | 0.052 | 0.374 | 4.44 | ± | 1.62 | 4.30 |
| **CCL-15** | 259.62 | ± | 76.37 | 243.61 | 0.07 | 0.48 | 229.17 | ± | 72.74 | 217.03 |
| **IL-8** | 13.74 | ± | 3.45 | 13.50 | 0.09 | 0.52 | 18.91 | ± | 12.47 | 16.25 |
| **CXCL-9** | 8.50 | ± | 9.23 | 6.73 | 0.11 | 0.64 | 10.28 | ± | 7.84 | 9.02 |
| **sCD30** | 340.97 | ± | 125.74 | 308.24 | 0.13 | 0.64 | 292.69 | ± | 143.52 | 257.68 |
| **IFN-γ** | 2.90 | ± | 1.15 | 2.69 | 0.15 | 0.64 | 2.32 | ± | 1.13 | 2.31 |
| **CCL-19** | 29.91 | ± | 14.47 | 28.84 | 0.17 | 0.64 | 39.10 | ± | 21.42 | 33.87 |
| **Osteocalcin** | 294.27 | ± | 124.62 | 277.86 | 0.17 | 0.64 | 254.43 | ± | 150.23 | 224.08 |
| **CCL-11** | 1.27 | ± | 0.44 | 1.07 | 0.17 | 0.64 | 1.13 | ± | 0.53 | 1.03 |
| **CCL-23** | 3.99 | ± | 5.24 | 2.71 | 0.17 | 0.64 | 3.74 | ± | 1.94 | 3.28 |
| **IL-34** | 113.34 | ± | 119.24 | 74.22 | 0.18 | 0.64 | 99.66 | ± | 43.35 | 91.87 |
| **sTNF-R2** | 314.01 | ± | 175.33 | 272.15 | 0.20 | 0.67 | 418.51 | ± | 247.52 | 334.57 |
| **CXCL-6** | 2.60 | ± | 4.83 | 1.71 | 0.24 | 0.76 | 1.99 | ± | 1.02 | 1.96 |
| **Pentraxin-3** | 23.61 | ± | 10.69 | 20.75 | 0.25 | 0.76 | 20.29 | ± | 3.66 | 19.47 |
| **MCP-2 CCL8** | 3.84 | ± | 2.11 | 3.36 | 0.30 | 0.86 | 4.48 | ± | 2.51 | 3.84 |
| **G-CSF** | 14.15 | ± | 6.79 | 15.19 | 0.33 | 0.92 | 13.92 | ± | 7.44 | 13.05 |
| **CCL-26** | 1.37 | ± | 2.00 | 0.86 | 0.37 | 0.93 | 0.95 | ± | 0.24 | 0.92 |
| **IL-26** | 41.77 | ± | 17.66 | 38.23 | 0.39 | 0.93 | 37.31 | ± | 15.85 | 35.52 |
| **IL-19** | 9.72 | ± | 7.89 | 7.79 | 0.42 | 0.93 | 7.52 | ± | 4.34 | 7.20 |
| **IL-2** | 1.31 | ± | 3.39 | 0.78 | 0.45 | 0.93 | 0.66 | ± | 0.52 | 0.82 |
| **CX3CL-1** | 33.37 | ± | 16.02 | 31.02 | 0.45 | 0.93 | 34.58 | ± | 11.24 | 32.25 |
| **sCD163** | 7,796.10 | ± | 2,973.11 | 6,708.03 | 0.45 | 0.93 | 9,027.29 | ± | 3,886.32 | 8,520.16 |
| **IL-22** | 6.12 | ± | 8.42 | 3.60 | 0.47 | 0.93 | 4.30 | ± | 2.92 | 3.89 |
| **IL-27** | 18.39 | ± | 19.72 | 14.23 | 0.48 | 0.93 | 13.35 | ± | 8.53 | 13.54 |
| **TNF-α** | 10.75 | ± | 7.82 | 9.36 | 0.51 | 0.93 | 8.75 | ± | 4.83 | 9.04 |
| **IL-15** | 30.33 | ± | 14.59 | 33.47 | 0.52 | 0.93 | 27.58 | ± | 15.86 | 27.88 |
| **CXCL-1** | 34.34 | ± | 14.75 | 30.10 | 0.53 | 0.93 | 33.17 | ± | 6.75 | 32.58 |
| **IL-11** | 1.52 | ± | 2.12 | 1.04 | 0.54 | 0.93 | 0.96 | ± | 0.65 | 0.87 |
| **CCL-21** | 181.26 | ± | 72.22 | 152.16 | 0.54 | 0.93 | 182.02 | ± | 56.26 | 181.52 |
| **IL-9** | 6.85 | ± | 2.83 | 6.54 | 0.54 | 0.93 | 6.39 | ± | 3.48 | 5.81 |
| **LIGHT TNFSF14** | 191.26 | ± | 64.32 | 214.73 | 0.56 | 0.93 | 188.36 | ± | 66.17 | 206.20 |
| **IL-1Ra** | 141.31 | ± | 57.31 | 143.88 | 0.57 | 0.93 | 133.41 | ± | 60.66 | 118.23 |
| **MIP-1β** | 3.55 | ± | 1.49 | 3.34 | 0.59 | 0.93 | 3.74 | ± | 1.39 | 3.50 |
| **Osteopontin** | 24,433.00 | ± | 6,384.84 | 23,990.75 | 0.59 | 0.93 | 27,497.95 | ± | 14,475.06 | 24,671.33 |
| **PDGF-BB** | 4.72 | ± | 5.04 | 3.31 | 0.60 | 0.93 | 4.02 | ± | 4.74 | 1.66 |
| **APRIL** | 19,024.94 | ± | 15,387.22 | 17,134.52 | 0.61 | 0.93 | 15,645.68 | ± | 10,477.43 | 14,166.91 |
| **TNFSF-12** | 1,042.37 | ± | 392.83 | 901.15 | 0.62 | 0.93 | 940.29 | ± | 249.66 | 896.88 |
| **RANTES** | 5.67 | ± | 1.88 | 5.12 | 0.65 | 0.93 | 6.21 | ± | 2.61 | 5.47 |
| **VEGF** | 143.24 | ± | 60.59 | 134.80 | 0.66 | 0.93 | 134.61 | ± | 61.02 | 121.84 |
| **GM-CSF** | 45.19 | ± | 21.52 | 42.59 | 0.68 | 0.93 | 41.69 | ± | 7.13 | 43.06 |
| **IL-35** | 43.25 | ± | 105.33 | 18.07 | 0.71 | 0.93 | 19.80 | ± | 25.14 | 16.99 |
| **CCL-7** | 14.14 | ± | 30.38 | 7.13 | 0.72 | 0.93 | 7.46 | ± | 2.64 | 7.13 |
| **CCL25** | 32.90 | ± | 46.60 | 22.10 | 0.72 | 0.93 | 22.82 | ± | 5.02 | 22.84 |
| **sIL-6Rb** | 24,145.20 | ± | 9,000.37 | 24,953.25 | 0.72 | 0.93 | 25,086.75 | ± | 10,078.19 | 24,404.04 |
| **IL-32** | 21.57 | ± | 13.73 | 20.23 | 0.73 | 0.93 | 18.79 | ± | 6.62 | 19.21 |
| **IL-4** | 8.14 | ± | 6.81 | 5.60 | 0.73 | 0.93 | 6.70 | ± | 3.98 | 7.39 |
| **IL-10** | 7.20 | ± | 3.00 | 6.08 | 0.74 | 0.93 | 6.89 | ± | 1.79 | 6.78 |
| **IL-20** | 45.42 | ± | 54.67 | 25.87 | 0.75 | 0.93 | 32.09 | ± | 14.75 | 27.18 |
| **IFN-β** | 20.20 | ± | 21.92 | 14.44 | 0.77 | 0.93 | 15.04 | ± | 10.09 | 15.60 |
| **TSLP** | 12.26 | ± | 20.34 | 6.24 | 0.77 | 0.93 | 7.50 | ± | 5.07 | 6.16 |
| **CCL-24** | 27.83 | ± | 7.62 | 25.63 | 0.82 | 0.94 | 26.79 | ± | 2.99 | 27.07 |
| **sTNF-R1** | 1,533.24 | ± | 412.07 | 1,529.34 | 0.84 | 0.94 | 1,561.50 | ± | 403.87 | 1,545.38 |
| **CXCL-25** | 124.12 | ± | 168.53 | 90.09 | 0.86 | 0.94 | 92.91 | ± | 28.68 | 90.53 |
| **CXCL-16** | 575.37 | ± | 46.14 | 580.46 | 0.86 | 0.94 | 577.77 | ± | 49.95 | 581.35 |
| **CXCL-2** | 6.78 | ± | 12.67 | 4.24 | 0.87 | 0.94 | 4.32 | ± | 3.29 | 4.10 |
| **Q_alb_** | 4.84 | ± | 1.79 | 4.38 | 0.88 | 0.94 | 5.01 | ± | 1.86 | 4.44 |
| **MIF** | 1,298.60 | ± | 1,448.74 | 751.00 | 0.88 | 0.94 | 1,127.14 | ± | 1,043.77 | 877.34 |
| **CCL-13** | 0.67 | ± | 0.64 | 0.49 | 0.89 | 0.94 | 0.54 | ± | 0.16 | 0.51 |
| **CXCL-12** | 3,329.42 | ± | 1,114.73 | 3,064.33 | 0.93 | 0.97 | 3,391.48 | ± | 1,188.34 | 3,447.17 |
| **sIL-6Ra** | 1,086.98 | ± | 434.93 | 934.83 | 0.95 | 0.97 | 1,092.84 | ± | 401.16 | 1,033.00 |
| **IL-6** | 4.33 | ± | 3.65 | 2.70 | 0.96 | 0.97 | 5.16 | ± | 8.33 | 3.03 |
