# Supplementary material for: Wide Cytokine Analysis in Cerebrospinal Fluid at Diagnosis Identified CCL-3 as a Possible Prognostic Factor for Multiple Sclerosis
Source: Front Immunol. 2020 Mar 5;11:174. doi: 10.3389/fimmu.2020.00174 (PMC7066207; doi:10.3389/fimmu.2020.00174)
Supplement: Supplementary file 3 [file Data_Sheet_3.DOCX]

**Supplementary Materials 3. CCL-2 and CCL-3 associate with MS diagnosis.** Univariate and multivariate analysis assessing the effect of cytokines on the diagnosis of RMS.

|  | **Univariate Analysis** | | **Multivariate Analysis** | |
| --- | --- | --- | --- | --- |
|  | O.R. (95%CI) | p-value | O.R. (95%CI) | p-value |
| APRIL | 1.00 | 0.318 |  |  |
| BAFF | 1.00 | 0.041 |  |  |
| CCL-1 | 0.97 | 0.745 |  |  |
| CCL-15 | 1.00 | 0.151 |  |  |
| CCL-19 | 1.03 | 0.111 |  |  |
| CCL-21 | 1.00 | 0.965 |  |  |
| CCL-22 | 1.19 | 0.007 |  |  |
| CCL-23 | 0.98 | 0.780 |  |  |
| CCL-24 | 0.96 | 0.437 |  |  |
| CCL-25 | 0.98 | 0.307 |  |  |
| CCL-26 | 0.69 | 0.296 |  |  |
| CCL-27 | 0.73 | 0.045 |  |  |
| CX3CL-1 | 1.01 | 0.731 |  |  |
| CXCL-1 | 0.99 | 0.658 |  |  |
| CXCL-10 | 1.01 | 0.019 |  |  |
| CXCL-11 | 2.74 | 0.153 |  |  |
| CXCL-12 | 1.00 | 0.849 |  |  |
| CXCL-13 | 3.23 | 0.006 |  |  |
| CXCL-16 | 1.00 | 0.860 |  |  |
| CXCL-2 | 0.96 | 0.287 |  |  |
| CXCL-25 | 1.00 | 0.302 |  |  |
| CXCL-9 | 1.03 | 0.443 |  |  |
| CXCL-6 | 0.93 | 0.438 |  |  |
| CCL-11 | 0.58 | 0.317 |  |  |
| GM-CSF | 0.98 | 0.354 |  |  |
| G-CSF | 1.00 | 0.908 |  |  |
| IFN-β | 0.98 | 0.231 |  |  |
| IFN-γ | 0.63 | 0.082 |  |  |
| IL-1-Ra | 1.00 | 0.636 |  |  |
| IL-10 | 0.94 | 0.612 |  |  |
| IL-11 | 0.70 | 0.191 |  |  |
| IL-15 | 0.99 | 0.527 |  |  |
| IL-16 | 1.01 | 0.418 |  |  |
| IL-19 | 0.93 | 0.182 |  |  |
| IL-2 | 0.83 | 0.298 |  |  |
| IL-20 | 0.99 | 0.210 |  |  |
| IL-22 | 0.94 | 0.243 |  |  |
| IL-26 | 0.98 | 0.334 |  |  |
| IL-27 | 0.97 | 0.191 |  |  |
| IL-32 | 0.97 | 0.292 |  |  |
| IL-34 | 1.00 | 0.502 |  |  |
| IL-35 | 0.99 | 0.232 |  |  |
| IL-4 | 0.94 | 0.301 |  |  |
| IL-6 | 1.02 | 0.693 |  |  |
| IL-8 | 1.13 | 0.091 |  |  |
| IL-9 | 0.96 | 0.619 |  |  |
| LIGHT-TNFSF14 | 1.00 | 0.874 |  |  |
| CCL-2 | 0.99 | 0.007 | 0.98 | 0.001 |
| CCL-8 | 1.13 | 0.348 |  |  |
| CCL-7 | 0.97 | 0.325 |  |  |
| CCL-13 | 0.39 | 0.272 |  |  |
| MIF | 1.00 | 0.598 |  |  |
| CCL-3 | 63.95 | 0.015 | >999.999 | 0.0008 |
| MIP-1β | 1.10 | 0.631 |  |  |
| Osteocalcin | 1.00 | 0.331 |  |  |
| Osteopontin | 1.00 | 0.401 |  |  |
| PDGF-BB | 0.97 | 0.601 |  |  |
| Pentraxin-3 | 0.92 | 0.144 |  |  |
| Q_alb_ | 1.06 | 0.736 |  |  |
| RANTES | 1.10 | 0.433 |  |  |
| TNFSF-12 | 1.00 | 0.223 |  |  |
| TNF-α | 0.94 | 0.228 |  |  |
| TSLP | 0.97 | 0.220 |  |  |
| VEGF | 1.00 | 0.611 |  |  |
| sCD163 | 1.00 | 0.240 |  |  |
| sCD30 | 1.00 | 0.225 |  |  |
| sIL-6Ra | 1.00 | 0.959 |  |  |
| sIL-6Rb | 1.00 | 0.731 |  |  |
| sTNF-R1 | 1.00 | 0.802 |  |  |
| sTNF-R2 | 1.00 | 0.120 |  |  |
