# Supplementary material for: Wide Cytokine Analysis in Cerebrospinal Fluid at Diagnosis Identified CCL-3 as a Possible Prognostic Factor for Multiple Sclerosis
Source: Front Immunol. 2020 Mar 5;11:174. doi: 10.3389/fimmu.2020.00174 (PMC7066207; doi:10.3389/fimmu.2020.00174)
Supplement: Supplementary file 4 [file Data_Sheet_4.DOCX]

**Supplementary Materials 4. *BAFF and CCL-3*** **associate with both quantitative and qualitative markers of IgG intrathecal synthesis.** Univariate and multivariate analysis assessing the effect of cytokines on these markers.

|  | **IgGIF** | | | | | | | | **IgGOB** | | | | | | | |
| --- | --- | --- | --- | --- | --- | --- | --- | --- | --- | --- | --- | --- | --- | --- | --- | --- |
| **Effect** | **Odds Ratio** | **LCL** | **UCL** | **p-value** | **Odds Ratio** | **LCL** | **UCL** | **p-value** | **Odds Ratio** | **LCL** | **UCL** | **p-value** | **Odds Ratio** | **LCL** | **UCL** | **p-value** |
| MIP-1α | 237,431.00 | 8,601.00 | >999.999 | 0.0012 | >999.9 | 88998.0 | >999.9 | 0.0037 | 7.53 | 1.12 | 50.6 | 0.0378 | >999.9 | 8.186 | >999.9 | 0.021 |
| BAFF | 0.999 | 0.999 | 1.00 | 0.0071 | 0.999 | 0.999 | 1.00 | 0.008 | 0.999 | 0.998 | 1.00 | 0.0004 | 0.999 | 0.998 | 1.00 | 0.039 |
| sCD163 | 1.00 | 1.00 | 1.00 | 0.0281 | 1.00 | 1.00 | 1.00 | 0.018 | 1.00 | 1.00 | 1.00 | 0.9695 |  |  |  |  |
| IL-15 | 0.965 | 0.932 | 0.999 | 0.0451 | 0.89 | 0.82 | 0.98 | 0.017 | 0.963 | 0.927 | 1.00 | 0.051 |  |  |  |  |
| CCL-22 | 1,123.00 | 1,036.00 | 1,217.00 | 0.0046 |  |  |  |  | 1,047.00 | 1,008.00 | 1,088.00 | 0.0178 | 1,047.00 | 1,008.00 | 1,088.00 | 0.04 |
| Pentraxin-3 | 0.946 | 0.857 | 1,046.00 | 0.2786 |  |  |  |  | 0.629 | 0.468 | 0.845 | 0.0021 | 0.5 | 0.27 | 0.94 | 0.032 |
| APRIL | 1.00 | 1.00 | 1.00 | 0.9236 |  |  |  |  | 1.00 | 1.00 | 1.00 | 0.0844 |  |  |  |  |
| CCL-1 | 1,007.00 | 0.863 | 1,176.00 | 0.9283 |  |  |  |  | 1,007.00 | 0.858 | 1,182.00 | 0.9321 |  |  |  |  |
| CCL-15 | 0.998 | 0.991 | 1,005.00 | 0.5556 |  |  |  |  | 0.99 | 0.981 | 0.999 | 0.0227 |  |  |  |  |
| CCL-19 | 1,023.00 | 0.996 | 1,051.00 | 0.0926 |  |  |  |  | 1,025.00 | 0.998 | 1,053.00 | 0.068 |  |  |  |  |
| CCL-21 | 0.995 | 0.986 | 1,003.00 | 0.212 |  |  |  |  | 0.999 | 0.99 | 1,008.00 | 0.7995 |  |  |  |  |
| CCL-23 | 1.02 | 0.865 | 1,202.00 | 0.813 |  |  |  |  | 1,094.00 | 0.919 | 1,303.00 | 0.3124 |  |  |  |  |
| CCL-24 | 0.945 | 0.839 | 1,065.00 | 0.3527 |  |  |  |  | 0.938 | 0.807 | 1,089.00 | 0.3997 |  |  |  |  |
| CCL-25 | 0.99 | 0.963 | 1,017.00 | 0.4592 |  |  |  |  | 0.96 | 0.857 | 1,074.00 | 0.472 |  |  |  |  |
| CCL-26 | 0.818 | 0.461 | 1,451.00 | 0.4921 |  |  |  |  | 0.819 | 0.36 | 1,865.00 | 0.6347 |  |  |  |  |
| CCL-27 | 0.87 | 0.696 | 1,088.00 | 0.2224 |  |  |  |  | 0.871 | 0.661 | 1,147.00 | 0.3262 |  |  |  |  |
| CX3CL-1 | 1,011.00 | 0.971 | 1,053.00 | 0.5993 |  |  |  |  | 0.966 | 0.918 | 1,016.00 | 0.1769 |  |  |  |  |
| CXCL-1 | 0.994 | 0.944 | 1,048.00 | 0.8322 |  |  |  |  | 1,008.00 | 0.954 | 1,064.00 | 0.7831 |  |  |  |  |
| CXCL-10 | 1,006.00 | 1001.00 | 1,011.00 | 0.0124 |  |  |  |  | 1,002.00 | 0.999 | 1,005.00 | 0.1879 |  |  |  |  |
| CXCL-11 | 3,202.00 | 0.978 | 10,484.00 | 0.0545 |  |  |  |  | 2,916.00 | 1,047.00 | 8,121.00 | 0.0406 |  |  |  |  |
| CXCL-12 | 1.00 | 0.999 | 1.00 | 0.7259 |  |  |  |  | 1.00 | 1.00 | 1001.00 | 0.7049 |  |  |  |  |
| CXCL-13 | 1,493.00 | 1129.00 | 1,974.00 | 0.005 |  |  |  |  | 1,076.00 | 1,006.00 | 1.15 | 0.0328 |  |  |  |  |
| CXCL-16 | 1,004.00 | 0.994 | 1,014.00 | 0.458 |  |  |  |  | 0.995 | 0.984 | 1006.00 | 0.3353 |  |  |  |  |
| CXCL-2 | 0.966 | 0.888 | 1,052.00 | 0.4249 |  |  |  |  | 0.982 | 0.895 | 1,077.00 | 0.6948 |  |  |  |  |
| CXCL-25 | 0.998 | 0.993 | 1,004.00 | 0.6142 |  |  |  |  | 0.994 | 0.978 | 1.01 | 0.4683 |  |  |  |  |
| CXC-L9 | 1,036.00 | 0.967 | 1.11 | 0.3102 |  |  |  |  | 1,009.00 | 0.947 | 1,075.00 | 0.7871 |  |  |  |  |
| CXCL-6 | 0.99 | 0.819 | 1,198.00 | 0.9201 |  |  |  |  | 0.979 | 0.784 | 1,223.00 | 0.8516 |  |  |  |  |
| CCL-11 | 0.611 | 0.227 | 1,644.00 | 0.3293 |  |  |  |  | 0.155 | 0.033 | 0.728 | 0.0182 |  |  |  |  |
| GM-CSF | 0.982 | 0.939 | 1,027.00 | 0.422 |  |  |  |  | 0.97 | 0.908 | 1,037.00 | 0.3726 |  |  |  |  |
| G-CSF | 0.98 | 0.915 | 1,051.00 | 0.573 |  |  |  |  | 0.901 | 0.819 | 0.991 | 0.0311 |  |  |  |  |
| IFN-β | 1,008.00 | 0.971 | 1,046.00 | 0.6759 |  |  |  |  | 0.973 | 0.922 | 1,027.00 | 0.3219 |  |  |  |  |
| IFN-γ | 0.549 | 0.332 | 0.906 | 0.0189 |  |  |  |  | 0.518 | 0.299 | 0.897 | 0.0188 |  |  |  |  |
| IL1-Ra | 0.995 | 0.987 | 1,004.00 | 0.2509 |  |  |  |  | 0.989 | 0.978 | 1.00 | 0.052 |  |  |  |  |
| IL-10 | 0.984 | 0.782 | 1,237.00 | 0.8879 |  |  |  |  | 0.973 | 0.757 | 1.25 | 0.8314 |  |  |  |  |
| IL-11 | 0.958 | 0.641 | 1,433.00 | 0.8348 |  |  |  |  | 0.745 | 0.376 | 1,474.00 | 0.3978 |  |  |  |  |
| IL-16 | 1,006.00 | 0.995 | 1,018.00 | 0.2757 |  |  |  |  | 1,002.00 | 0.993 | 1,011.00 | 0.691 |  |  |  |  |
| IL-19 | 1,003.00 | 0.917 | 1,097.00 | 0.9496 |  |  |  |  | 0.928 | 0.822 | 1,048.00 | 0.2282 |  |  |  |  |
| IL-2 | 0.894 | 0.647 | 1,237.00 | 0.5001 |  |  |  |  | 0.816 | 0.416 | 1,602.00 | 0.5548 |  |  |  |  |
| IL-20 | 0.996 | 0.98 | 1,013.00 | 0.6295 |  |  |  |  | 0.977 | 0.937 | 1,019.00 | 0.2801 |  |  |  |  |
| IL-22 | 0.965 | 0.87 | 1,071.00 | 0.5062 |  |  |  |  | 0.864 | 0.695 | 1,075.00 | 0.1898 |  |  |  |  |
| IL-26 | 1,013.00 | 0.982 | 1,045.00 | 0.4187 |  |  |  |  | 1,001.00 | 0.969 | 1,034.00 | 0.952 |  |  |  |  |
| IL-27 | 0.996 | 0.958 | 1,036.00 | 0.851 |  |  |  |  | 0.968 | 0.911 | 1,028.00 | 0.2868 |  |  |  |  |
| IL-32 | 0.955 | 0.895 | 1.02 | 0.1705 |  |  |  |  | 0.926 | 0.85 | 1.01 | 0.0817 |  |  |  |  |
| IL-34 | 0.999 | 0.993 | 1,006.00 | 0.8773 |  |  |  |  | 0.995 | 0.984 | 1,007.00 | 0.3925 |  |  |  |  |
| IL-35 | 0.998 | 0.989 | 1,007.00 | 0.601 |  |  |  |  | 0.992 | 0.973 | 1,011.00 | 0.4073 |  |  |  |  |
| IL-4 | 1.04 | 0.936 | 1,156.00 | 0.4611 |  |  |  |  | 0.95 | 0.845 | 1,068.00 | 0.3925 |  |  |  |  |
| IL-6 | 1.06 | 0.949 | 1,184.00 | 0.2991 |  |  |  |  | 1,012.00 | 0.946 | 1,084.00 | 0.7254 |  |  |  |  |
| IL-8 | 1,082.00 | 0.986 | 1,188.00 | 0.0978 |  |  |  |  | 0.999 | 0.952 | 1,048.00 | 0.9594 |  |  |  |  |
| IL-9 | 0.895 | 0.764 | 1.05 | 0.1727 |  |  |  |  | 0.705 | 0.545 | 0.914 | 0.0082 |  |  |  |  |
| LIGHT-TNFSF14 | 0.993 | 0.985 | 1,001.00 | 0.0744 |  |  |  |  | 0.997 | 0.989 | 1,005.00 | 0.4497 |  |  |  |  |
| CCL2 | 0.994 | 0.988 | 1.00 | 0.0378 |  |  |  |  | 0.993 | 0.987 | 1.00 | 0.0488 |  |  |  |  |
| CCL8 | 1,228.00 | 0.967 | 1.56 | 0.0917 |  |  |  |  | 1.21 | 0.97 | 1,509.00 | 0.0917 |  |  |  |  |
| CCL7 | 0.982 | 0.938 | 1,029.00 | 0.4477 |  |  |  |  | 0.983 | 0.917 | 1,053.00 | 0.6233 |  |  |  |  |
| CCL13 | 0.667 | 0.154 | 2,882.00 | 0.5874 |  |  |  |  | 0.227 | 0.008 | 6,078.00 | 0.3765 |  |  |  |  |
| MIF | 1.00 | 0.999 | 1.00 | 0.1522 |  |  |  |  | 1.00 | 0.999 | 1.00 | 0.3212 |  |  |  |  |
| MIP-1β | 1049.00 | 0.737 | 1,493.00 | 0.79 |  |  |  |  | 1,067.00 | 0.733 | 1,553.00 | 0.736 |  |  |  |  |
| Osteocalcin | 0.999 | 0.996 | 1,003.00 | 0.6022 |  |  |  |  | 0.996 | 0.991 | 1,001.00 | 0.1062 |  |  |  |  |
| Osteopontin | 1.00 | 1.00 | 1.00 | 0.3793 |  |  |  |  | 1.00 | 1.00 | 1.00 | 0.3488 |  |  |  |  |
| PDGF-BB | 0.97 | 0.875 | 1,076.00 | 0.5667 |  |  |  |  | 0.91 | 0.803 | 1,032.00 | 0.143 |  |  |  |  |
| Q_alb_ | 1163.00 | 0.878 | 1,541.00 | 0.2918 |  |  |  |  | 0.562 | 0.367 | 0.862 | 0.0082 |  |  |  |  |
| RANTES | 0.978 | 0.799 | 1,198.00 | 0.8319 |  |  |  |  | 0.87 | 0.686 | 1,104.00 | 0.2521 |  |  |  |  |
| TNFSF-12 | 0.998 | 0.997 | 1.00 | 0.0966 |  |  |  |  | 0.998 | 0.996 | 1.00 | 0.1092 |  |  |  |  |
| TNF-α | 1024.00 | 0.938 | 1,119.00 | 0.5951 |  |  |  |  | 0.958 | 0.866 | 1.06 | 0.4043 |  |  |  |  |
| TSLP | 0.983 | 0.937 | 1,032.00 | 0.4916 |  |  |  |  | 0.938 | 0.817 | 1,079.00 | 0.371 |  |  |  |  |
| VEGF | 0.994 | 0.985 | 1,002.00 | 0.1376 |  |  |  |  | 0.991 | 0.982 | 1,001.00 | 0.082 |  |  |  |  |
| sCD30 | 0.999 | 0.996 | 1,003.00 | 0.7027 |  |  |  |  | 0.998 | 0.993 | 1,002.00 | 0.2604 |  |  |  |  |
| sIL-6Ra | 1.00 | 0.999 | 1,001.00 | 0.7326 |  |  |  |  | 1.00 | 0.998 | 1,001.00 | 0.5936 |  |  |  |  |
| sIL-6Rb | 1.00 | 1.00 | 1.00 | 0.9158 |  |  |  |  | 1.00 | 1.00 | 1.00 | 0.1443 |  |  |  |  |
| sTNF-R1 | 1001.00 | 1.00 | 1,002.00 | 0.2003 |  |  |  |  | 0.999 | 0.998 | 1.00 | 0.1662 |  |  |  |  |
| sTNF-R2 | 1004.00 | 1001.00 | 1,006.00 | 0.0091 |  |  |  |  | 1.00 | 0.998 | 1,002.00 | 0.8936 |  |  |  |  |
